# Defining the Optimal Dietary Approach for Safe, Effective and Sustainable Weight Loss in Overweight and Obese Adults

**DOI:** 10.3390/healthcare6030073

**Published:** 2018-06-28

**Authors:** Chrysi Koliaki, Theodoros Spinos, Μarianna Spinou, Μaria-Eugenia Brinia, Dimitra Mitsopoulou, Nicholas Katsilambros

**Affiliations:** 1 First Department of Propaedeutic Medicine, National Kapodistrian University of Athens, Laiko University Hospital, Athens 11527, Greece; nicholaskatsilambros@gmail.com; 2 Medical School, National Kapodistrian University of Athens, Athens 11527, Greece; thspinos@otenet.gr (T.S.); mspinou@otenet.gr (M.S.); mairyjane1054@gmail.com (M.-E.B.); dimits96@gmail.com (D.M.); 3 Research Laboratory Christeas Hall, Medical School, National Kapodistrian University of Athens, Athens 11527, Greece

**Keywords:** obesity, weight loss diets, macronutrient composition, safety, efficacy

## Abstract

Various dietary approaches with different caloric content and macronutrient composition have been recommended to treat obesity in adults. Although their safety and efficacy profile has been assessed in numerous randomized clinical trials, reviews and meta-analyses, the characteristics of the optimal dietary weight loss strategy remain controversial. This mini-review will provide general principles and practical recommendations for the dietary management of obesity and will further explore the components of the optimal dietary intervention. To this end, various dietary plans are critically discussed, including low-fat diets, low-carbohydrate diets, high-protein diets, very low-calorie diets with meal replacements, Mediterranean diet, and diets with intermittent energy restriction. As a general principle, the optimal diet to treat obesity should be safe, efficacious, healthy and nutritionally adequate, culturally acceptable and economically affordable, and should ensure long-term compliance and maintenance of weight loss. Setting realistic goals for weight loss and pursuing a balanced dietary plan tailored to individual needs, preferences, and medical conditions, are the key principles to facilitate weight loss in obese patients and most importantly reduce their overall cardiometabolic risk and other obesity-related comorbidities.

## 1. Introduction

Identifying safe and effective strategies for long-term weight control is critical to reduce the alarming prevalence of overweight and obesity in adults and adolescents worldwide and mitigate obesity-associated health risks [1]. Obesity and overweight affect together over a third of the world’s population today, and if current trends continue, an estimated 38% of the world’s adult population will be overweight and another 20% will be obese by 2030 [2]. Although obesity is a complex and multifactorial disease with genetic, behavioral, socioeconomic, and environmental origins, it is also preventable and treatable to a great extent [3]. There is no doubt that the first-line treatment of obesity is dietary management combined with behavior modification, and secondarily, increased physical activity [4]. Weight loss medication and bariatric surgery are further recommended for specific subgroups of obese patients [5]. 

Dietary guidelines for weight loss vary greatly between different scientific societies and have been revised many times, reflecting the uncertainty in the field of nutritional management of obesity and the difficulty to generate uniform recommendations for all patients [5,6]. Although there are dozens of weight loss diets promising to reduce body weight [7], the characteristics of the optimal strategy remain controversial, and no single dietary strategy is uniformly superior to others in terms of weight loss and maintenance for the general population.

The optimal macronutrient ratio of a diet, or else the proportion of calories contributed by fat, carbohydrate, and protein, has received significant attention in the past decades for its potential relevance in weight loss [8], but remains still elusive. Some researchers emphasize the role of energy deficit irrespective of macronutrient composition [9], some others highlight the role of macronutrient composition irrespective of caloric count [10,11], and finally others underscore the role of diet quality by means of naturally cooked and unprocessed healthy foods irrespective of macronutrient composition or energy intake [12,13], to achieve weight loss and other health benefits. All these approaches have their own rationale and are all evidence-based and partly correct. However, the key to successful weight loss lies in the prudent combination of all these approaches in the context of a healthy and balanced diet without severe restrictions or nutritional exaggerations. 

The present review aims to provide general principles and practical recommendations for the dietary management of obesity, and further explore the components of the optimal dietary intervention. To this end, various dietary plans are critically discussed, such as low-fat diets, low-carbohydrate diets, high-protein diets, formula diets, Mediterranean diet, and diets with intermittent energy restriction, to define the optimal dietary approach for a safe, effective, and sustainable weight loss in overweight and obese adults. 

## 2. General Principles

Generally, it is not possible to lose weight without a negative energy balance [14]. It is, therefore, necessary that energy intake is consistently lower than energy expenditure to achieve weight loss [14]. In addition to energy restriction, the macronutrient composition of a diet was originally thought to play an important role for weight loss, on the grounds that diets with specific macronutrient ratios may be more appropriate to facilitate weight loss than others based on their differential potential to promote satiety, burn fat, and preserve metabolically active lean body mass [15]. However, the relevance of macronutrient-centered weight loss diets has been mainly substantiated by short-term studies [16,17,18], and results may be influenced by inter-individual biological and behavioral differences as well as different adherence rates. Of note, longer-term studies fail to provide any robust evidence in support of modulating dietary macronutrient composition to achieve a better weight loss outcome [19,20]. Although literature in the field remains inconclusive, the current state of evidence suggests that modification of dietary macronutrient composition is not as effective and clinically relevant for long-term weight management as originally believed [8,21]. 

Setting realistic goals for weight loss is extremely important since the adoption of strict and difficult to reach goals may often lead to failure and discouragement [22]. Aiming to lose 5–10% of initial body weight within the first six months is a realistic approach, which is furthermore paralleled by a significant improvement in cardiometabolic risk factors [5,6]. Beyond setting realistic goals, long-term adherence to dietary interventions represents also a great challenge, since many diets are pursued for only short periods of time-especially those with extreme restrictions-, leading to suboptimal long-term weight control [23]. 

An even more important goal than weight loss is weight loss maintenance and prevention of weight regain. The physiological response to weight loss is resistance to further weight loss through a compensational biological adaptation expressed as a shift in hormone balance related to appetite regulation, a decline of resting energy expenditure and a reduction of diet-induced thermogenesis [24,25]. Diet-induced weight loss has been associated with increased levels of orexigenic hormones (ghrelin) and reduced levels of anorexigenic hormones (leptin, peptide YY, cholecystokinin) [26]. The effects of weight loss on postprandial secretion of gastrointestinal satiety hormones remain controversial [26]. Furthermore, it has been shown that a weight loss of 10% may lead to a reduction of total energy expenditure by 550 kcal/d [27]. Among all the implicated mechanisms, the increased drive to eat after weight loss is, in fact, several-fold larger than the corresponding adaptations in total energy expenditure, and potentially represents the main driver of weight regain [28,29]. Of note, these adaptations may persist for up to a year after the initial weight loss and may often lead to relapse [30]. As a result, only around 20% of obese patients can preserve and stabilize the weight loss effect in the long-term (data from National Weight Control Registry) [31]. In detail, more than half of dieters regain most of their weight loss within the first 12 months and less than one-third can avoid weight regain over a three-year period [32,33]. 

The ideal weight loss maintenance diet should be continuous, easy to comply with, and of low energy density [34,35]. Predictors of successful long-term weight maintenance after initial weight loss involve frequent self-monitoring of body weight [36], medical supervision for psychological support and positive feedback [37], consistency of food intake [38], eating breakfast [39,40], low-fat intake [35], low intake of unhealthy snacks [41], and high levels of regular physical activity [42]. It has been further suggested by preliminary evidence that the space of a meal consumption (fast vs. slow) may also affect body weight control and maintenance [43]. It has been shown in healthy volunteers that consuming slowly a standardized meal may lead to a sharper rise in anorexigenic hormones and promote more a feeling of fullness compared to a faster rate of meal consumption [44]. These preliminary findings warrant, however, further investigation. 

## 3. Conventional Hypocaloric Diets

Conventional hypocaloric diets typically aim at reducing daily energy intake by 500–750 kcal. This energy restriction is usually achieved by diets of 1200–1500 kcal/d for females and 1500–1800 kcal/d for males [45]. Conventional diets are generally low-fat diets and most of them have the following macronutrient composition: 30% fat, 50% carbohydrate and 20% protein [45]. A particular emphasis is placed on reducing intake of saturated (animal-derived) fat and increasing intake of fiber-rich foods such as fruit and vegetables [45]. The latter can both promote satiety and provide a great variety of beneficial micronutrients. Reducing daily energy intake by 500–600 kcal can lead to a modest weight loss of approximately 0.5 kg per week or else 2 kg per month. This weight loss is usually seen only in the first months, since the rate of weight loss is expected to slow down because of the hormonal adaptations resisting weight loss, as described above [24]. It is essential that conventional diets are individualized based on the weight loss course of each subject, and individual food preferences need to be considered, since these diets are usually followed for long periods of time to achieve a clinically meaningful weight loss [45]. Although energy-restricted diets are modestly effective for short-term weight loss [46], individual response to hypocaloric diets is heterogeneous and long-term adherence to these diets is difficult to accomplish [47]. 

## 4. Low-Fat Diets

Low-fat diets have been recommended as safe and effective weight loss strategies for many decades on the basis of several observations: (1) energy from fat is less satiating than energy from carbohydrate, and a high fat/carbohydrate ratio in the diet can promote passive overconsumption, positive energy balance and weight gain in susceptible individuals [48,49,50]; (2) fat is more readily absorbed from the intestine than carbohydrate and fecal energy loss is much lower with a high dietary fat/carbohydrate ratio; (3) carbohydrate is more thermogenic than fat and energy expenditure is lower during a diet with a high fat/carbohydrate ratio than during a diet with a low fat/carbohydrate ratio [51,52]; and (4) a high-fat diet may damage the intestinal barrier and cause intestinal dysbiosis with an adverse impact on body weight and metabolic variables [53]. Another reason for lowering the proportion of calories consumed from fat is that a single gram of fat contains more than twice the calories of a gram of carbohydrates or protein (9 kcal/gram vs. 4 kcal/gram). Thus, reducing total fat intake may theoretically lead to a considerable effect on total amount of calories consumed. 

Despite the above theoretical considerations, randomized trials have failed to consistently demonstrate that reducing fat intake may be superior to other dietary interventions in terms of long-term weight loss. In a meta-analysis comparing several popular weight loss diets, low-fat diets were found to be equally effective as other diets in terms of weight loss, without however reporting any differences between diets in qualitative aspects, compliance rates and adverse events [54]. Another study has shown that both low-fat and higher-fat diets have similar effects on weight loss, total and visceral fat loss, and lean body mass preservation [8]. In this study, both diets were characterized by low intake of saturated fat and foods of high glycemic index and an increased intake of fiber-rich foods, suggesting that when standards of a high-quality diet are met, variations in macronutrient composition play a secondary role for weight loss [8]. In another systematic review and meta-analysis comparing low-fat diets with other dietary interventions, it was found that the long-term effect of low-fat diets on body weight depends primarily on the intensity of diet intervention in the comparison group [55]. When compared to usual diets, low-fat diets are indeed more effective in weight reduction with a slight to modest effect [55]. However, when compared to other higher-fat dietary interventions of similar intensity such as low-carbohydrate diets and especially when high adherence rates are achieved, low-fat diets are equally or less effective in achieving significant long-term weight control [55]. 

## 5. Low-Carbohydrate Diets

Low-carbohydrate diets originated from the old ketogenic Atkins diet [56], which was based on a severe restriction of carbohydrate intake (<30 g/d) and was characterized thus by poor dietary quality [57]. In the current versions of low-carbohydrate diets, carbohydrate restrictions are less severe compared to Atkins diet but still quite substantial, and there is also an increased fiber intake [45]. Long-term compliance to low-carbohydrate diets is both difficult and potentially hazardous since a significant reduction of carbohydrate intake in combination with high fat intake may lead to increased low-density lipoprotein (LDL) cholesterol levels and an elevated mortality risk [58]. Meta-analyses have shown that very low carbohydrate ketogenic diets are more effective short-term than other dietary strategies in terms of weight loss and improvement of metabolic variables in patients with diabetes [10]. Regarding low-carbohydrate diets, meta-analyses of randomized clinical trials provide conflicting evidence. Some meta-analyses have suggested that low-carbohydrate diets provide better weight loss outcomes than low-fat diets, but weight loss benefits should be weighed against potential risks associated with LDL-cholesterol increase [59]. On the other hand, other meta-analyses have shown that low-carbohydrate diets confer equal weight loss short-term as isoenergetic balanced [60] or low-fat diets [61]. Some other meta-analyses have suggested that low-carbohydrate diets without energy restriction are as effective as energy-restricted low-fat diets in weight loss, emphasizing potential favorable effects on triglycerides and high-density-lipoprotein (HDL) cholesterol levels [62]. Of note, data on the long-term (beyond one year) safety and efficacy of these diets are currently limited, so there is insufficient evidence to support their long-term use. 

A low-carbohydrate diet program has been recently introduced and successfully applied in Greece. This intervention is named Eurodiet and is based on the progressive reduction and reintroduction of carbohydrates in four consecutive stages [63]. At the last stage, patients apply a healthy balanced diet with characteristics of a Mediterranean diet and a modest restriction of carbohydrate intake. When this program is further accompanied by a frequent monitoring of psychological parameters and intense behavior modification, the effects are even greater [63]. 

## 6. High-Protein Diets

In high-protein diets, protein contributes by 20–30% to the total daily energy intake [64]. It has been suggested that such diets may deliver a greater weight loss than lower-protein diets (15–20%) due to their satiety-promoting effects and lean body mass preservation, in addition to relatively increased diet-induced thermogenesis [65,66]. Clinical intervention studies have shown that an ad libitum high-protein diet in overweight people may lead to a weight loss of 3.8 kg in a six-month weight loss program by enhancing satiety, as opposed to a high-carbohydrate diet [67]. It has been also demonstrated that an energy-restricted, high-protein diet may provide equal or even greater weight loss and metabolic benefits compared to a high-carbohydrate diet in obese women [68]. Furthermore, weight loss studies in overweight women have shown that diets with a high dietary protein to carbohydrate ratio may exert beneficial effects on body composition, blood lipid profile, and glucose homeostasis. These benefits may be partly mediated by effects on satiety and a lower glycemic load due to lower carbohydrate intake [69,70]. A higher protein intake during weight loss may also prevent some of the inevitable loss of lean body mass and may thus enhance insulin sensitivity [71,72], although this has not been observed at a very low energy intake [73]. In overweight men and women with either insulin resistance or type 2 diabetes, a high-protein weight loss diet (28–30% protein from mixed sources) was shown to enhance fat loss by 1–2 kg over 12 weeks, particularly in women, compared with an isocaloric high-carbohydrate diet [74,75]. Taken together, higher-protein diets may facilitate weight loss when compared to lower-protein diets in the short-term (up to 6 months), but longer-term data are limited and inconsistent [66]. The optimal amount and sources of dietary protein remain also controversial. Animal-derived proteins may be positively associated with obesity and weight gain as shown in longitudinal prospective studies, while plant-derived proteins may be protective for the development of obesity [19,20]. Furthermore, dietary patterns high in protein may vary in saturated fat and nutritional composition, and concerns have been raised regarding the effects of high-protein diets on serum lipids and subsequent cardiovascular disease risk [76]. In addition, high-protein diets and especially animal-derived proteins may pose an increased risk of nephrolithiasis, diabetes mellitus and atherosclerosis, as well as progressive kidney damage in susceptible individuals [77]. Based on the above, a prudent recommendation in dietary practice would be to partially replace refined carbohydrates with protein sources that are low in saturated fat [66]. An example of high-protein diets are formula diets, which are analytically described below. 

## 7. Formula Diets

Formula diets represent an additional evidence-based intervention in the weight management tool box. They provide a greater energy deficit than conventional hypocaloric diets and are also referred to as very-low-calorie diets (VLCD, <800 kcal/d) or low-calorie diets (LCD, 800–1200 kcal/d). They comprise ready-to-ingest meal replacements in the form of nutrient-enriched bars, soups, and drinks, which are low in carbohydrate and fat and rich in vitamins, minerals, and proteins of high biological value. As a result of drastic energy restriction, formula diets promote a substantial weight loss of 10–20 kg within 8–12 weeks [78]. After the initial phase of rapid weight loss, several strategies have been proposed to ensure weight loss maintenance and prevent weight regain, including high-protein diets, anti-obesity drugs, partial use of meal replacements, and most importantly, high levels of physical activity [79]. It has been shown that if the above strategies are pursued, weight loss maintenance is feasible after VLCDs [79]. After the desired weight loss is achieved, food is gradually reintroduced, and patients return to a healthy and balanced dietary plan. In pathophysiological terms, the reduced caloric and in particular the reduced carbohydrate content of formula diets increases circulating blood ketones and may thus reduce hyperinsulinemia of obese patients leading to sustained suppression of hunger and thus to better compliance [80]. The profound initial weight loss experienced by severely obese patients may further motivate them and promote thus adherence to the diet [78,81]. 

It is important to note that formula diets are intended for short-term use of maximum 12 weeks and should be always applied in carefully selected patients under continuous medical supervision and accompanied by sufficient education and psychological support. If the above requirements are met, formula diets may deliver significant weight loss and maintenance with concomitant health benefits in terms of metabolic profile and symptomatic improvement in several subgroups of patients, such as patients with diabetes [82], osteoarthritis [83], obstructive sleep apnea [84], psoriasis [85], and more commonly, in the pre-operative period of morbidly obese patients who are planned to undergo bariatric surgery, since the reduction of liver size achieved by VLCDs may facilitate surgical manipulations [86]. 

## 8. Mediterranean Diet

DIRECT study (Dietary Intervention Randomized Controlled Trial) compared a Mediterranean diet to a low-carbohydrate and a low-fat diet in 322 obese subjects (mean BMI 31 kg/m^2^, 86% males) in a controlled workplace setting [87]. At two years, mean weight loss was 2.9 kg for the low-fat, 4.4 kg for the Mediterranean diet and 4.7 kg for the low-carbohydrate group. Predictors of successful weight loss at six months were an increased intake of vegetables and a reduced intake of sweets and cakes [87]. At six years after study initiation, total weight loss was 0.6 kg in the low-fat, 3.1 kg in the Mediterranean diet and 1.7 kg in the low-carbohydrate group. Of note, Mediterranean and low-carbohydrate groups were not different from each other, but they were both superior to low-fat diet in terms of long-term weight maintenance [88]. In addition, it has been suggested that adherence to Mediterranean diet may be associated with reduced total and cause-specific mortality and promote thus longevity [89]. Furthermore, other data suggest that a diet supplemented by Mediterranean food products such as extra-virgin olive oil and nuts and resembling thus Mediterranean diet, may reduce the incidence of major cardiovascular events [90]. It may also reduce fasting glucose, lipids, and stroke incidence in a genetically susceptible high-risk population for cardiovascular events (PREDIMED study; PREvención con DIeta MEDiterránea) [91]. However, the cardioprotective properties of the Mediterranean diet need to be confirmed in other populations as well (outside Spain). Although epidemiological evidence regarding the overall health benefits of the Mediterranean diet is solid, the effect of this dietary pattern on long-term weight control remains to be investigated with additional randomized clinical trials conducted over longer periods of time in diverse cultures and populations. Furthermore, there is still no definitive data as to whether a specific component of Mediterranean diet is mainly responsible for the beneficial effects, or it is rather the combination and interaction of single ingredients which makes Mediterranean diet a healthy diet. 

## 9. Intermittent Diets

Intermittent energy restriction (IER) is based on the intermittent restriction of food intake, with shifts between periods of reduced energy intake and periods of unrestricted feeding [92,93]. The most commonly studied regimens of IER are those of energy restriction on two consecutive days per week, alternate day energy restriction by 60–70%, and complete fasting on alternate days (intermittent fasting, IF) [92]. Reviews in the field of intermittent dieting reveal the lack of high-quality evidence to support the superior or equal long-term safety and efficacy of intermittent diets compared to conventional diets with continuous energy restriction [94]. The few available randomized studies comparing intermittent with continuous hypocaloric diets in overweight and obese patients, report equal efficacy in terms of weight loss for a period up to 6 months [95,96,97]. To date, no studies suggest that intermittent diets can prevent weight gain in normal-weight individuals. Furthermore, data regarding the impact of intermittent diets on ectopic fat stores, adipocyte size and adipose tissue function, fat-free mass, insulin resistance and metabolic flexibility in humans, are scarce and heterogeneous [98]. Of note, some studies in animal models and normal-weight humans have shown detrimental effects of intermittent diets on metabolic homeostasis, raising safety concerns and the need for further investigation [96,99]. IER is generally preferable compared to complete intermittent fasting, due to its higher compliance, lower stress response and milder metabolic fluctuations (free fatty acid and ketone fluxes) [92]. 

At present, the optimal pattern and severity of energy restriction (e.g., two consecutive days per week i.e., 5:2, alternate days, five consecutive days per month, energy restriction by 60–70% or complete fasting) remains controversial. It remains also unclear which would be the optimal macronutrient composition of such intermittent diets. Taken together, in view of the multiple knowledge gaps and unaddressed questions in the field of intermittent dieting, the increasing popularity of these diets underscores the vital need for rigorous future research with appropriately designed, long-term, randomized studies in several subgroups of patients. In any case, an individualized critical appraisal is warranted to inform and guide the decision about which subjects might benefit from an intermittent diet for a short period of time, based on their social and personal contexts and coexisting clinical conditions. 

## 10. Diet-Induced Weight Loss: A Matter of Quality or Quantity?

The general rule that weight loss requires a negative energy balance with energy intake being lower than energy expenditure is both well-established and widely accepted. However, whether counting calories and limiting portion sizes is necessary to achieve weight loss has been disputed. The critical question of whether diet quantity or rather quality plays the most crucial role for long-term weight loss and maintenance was recently addressed in a randomized clinical trial (DIETFITS trial) conducted in Stanford University [13]. In this study, 609 overweight/obese and non-diabetic adults (age 18–50 years, BMI 28–40 kg/m^2^) were randomized to either a healthy low-fat or a healthy low-carbohydrate diet. All participants underwent an intensive dietician-guided training to eat healthy, minimally processed, whole foods cooked at home, without any caloric limits. The major finding of this study was that both diets delivered a significant weight loss of 5.3 kg for low-fat and 6 kg for low-carbohydrate over 12 months with similar improvement in waist circumference, body fat, fasting glucose and blood pressure. Interestingly, this effect was independent of genotype patterns or carbohydrate tolerance assessed by insulin secretion [13]. The authors concluded that diet quality defined as low intake of added sugars and highly processed foods and high intake of fruit, vegetables and whole-grain products, independent of energy intake, is fundamental for weight loss in overweight and obese adults, and furthermore, that predicting which diets are most effective based on genetics or insulin response to carbohydrates is not possible at the moment based on the limited number of candidate genes which were assessed [13]. Further research is warranted to explore in depth potential diet-genome interactions and possible epigenetic effects, to conclusively suggest that genetics or insulin response do not play a role in the effectiveness of particular diets. The finding that diet quality is essential for weight loss independent of energy intake is certainly stimulating, but several issues need to be considered. First, the long-term sustainability of newly-adopted dietary habits was not assessed in this study. Second, both groups ended up consuming fewer calories on average (daily energy deficit of 500–600 kcal) even though they were instructed not to count calories. Finally, the relatively modest weight loss effect of 6 kg within a year was not achieved without a cost, since multiple time-demanding educational sessions were required by highly-qualified healthcare professionals who emphasized behavior modification to support weight loss. 

## 11. Conclusions and Recommendations

The ideal diet for treatment of overweight and obesity is defined as being safe, efficacious, healthy, nutritionally adequate, culturally acceptable, and economically affordable. It should further ensure long-term compliance and maintenance of weight loss effect. Various dietary plans with different caloric content and macronutrient composition have been assessed in randomized clinical trials, reviews, and meta-analyses, and found to be promising in promoting weight loss in adults. Nevertheless, the optimal diet remains still under debate. Only general principles and recommendations can be provided, and no single diet can be prescribed to all people with obesity or recommended as the best fit-for-all diet without strict individualization. 

Conventional hypocaloric diets are safe, healthy, and modestly effective. There is insufficient evidence to support the superiority of low-fat diets over other higher-fat dietary interventions of similar intensity to achieve long-term weight loss and maintenance. Low-carbohydrate diets are effective and metabolically beneficial in the short-term, but long-term adherence is an issue. There might also be some health risks associated with long-term consumption of these diets, depending on their nutrient content as well as the individual’s health status and risk factor profile. High-protein diets may promote satiety and prevent loss of muscle mass but can be also difficult to adhere to in the long-term and potentially hazardous for subgroups of patients with impaired kidney function or other health problems. Formula diets are the most effective strategy to achieve substantial and rapid weight loss but are indicated for specific subgroups of patients and intended for short-term use. The Mediterranean diet is as effective as low-carbohydrate diets in weight loss and can also provide benefits for overall health due to its balanced composition and diversity of health-promoting micronutrients. Intermittent diets are promising, but long-term safety and efficacy data are lacking, and the optimal pattern and severity of energy restriction remain controversial. As to the question of whether diet quality or quantity is more important, energy intake does certainly play a role, but the most effective strategy to achieve long-term weight loss and good cardiometabolic health is shifting to a healthy dietary pattern, compatible with individual food preferences and lifestyle habits. This dietary pattern should have restrictions in added sugars, refined grains and highly processed foods and include instead fruit, vegetables, whole-grain foods, and low-fat dairy products, without necessarily counting calories daily. The dietary pattern described above should be further combined with intensive education, motivation, and behavior modification, to obtain slow but steady weight loss and other health benefits. 

Setting realistic goals for weight loss is important. Successful diets involve slow and steady changes. An even more important goal is weight loss maintenance and prevention of weight regain. The ideal weight loss maintenance diet should be continuous and easy to comply with. Eating high-quality fats and carbohydrates in the setting of a balanced diet cannot only promote weight loss, but also prevent coronary heart disease, diabetes, and other diseases. 

In general, scientific evidence about what constitutes a healthy diet is both consistent and straightforward: a healthy diet is a varied diet rich in fruits, vegetables, whole-grain products and high-quality proteins and poor in added sugar, refined grains, and highly-processed foods. People who make the above dietary choices may find it easier to control their body weight without necessarily counting calories or limiting portion sizes daily. Physical activity and energy expenditure play also an important role in weight loss since sedentary individuals need to reduce their energy intake even when consuming a healthy diet to achieve and maintain weight loss. Most importantly, the best diet is a diet that people can comply with for a long period of time without significant weight regain, so whatever facilitates this effort is greatly appreciable. 
